# The lifetime risk of developing type II diabetes in an urban community in Mumbai: findings from a ten-year retrospective cohort study

**DOI:** 10.1186/s12889-023-16596-6

**Published:** 2023-08-31

**Authors:** Palak Sharma, T. R. Dilip, Udaya Shankar Mishra, Anjali Kulkarni

**Affiliations:** 1 Department of Family and Generations, International Institute for Population Sciences, Mumbai, 400088 India; 2 Department of Bio-Statistics and Epidemiology, International Institute for Population Sciences, Mumbai, 400088 India; 3 Bhabha Atomic Research Center, Mumbai, 400088 India

**Keywords:** Diabetes, Lifetime risk, Cohort study, Electronic Medical Records (EMR)

## Abstract

**Background:**

Incidence and prevalence do not capture the risk of developing diabetes during a defined period and only limited evidence exists on the lifetime risk of diabetes based on longer and continuous follow-up studies in India. Lacunae in evidence on lifetime risk can be attributed primarily to the absence of comprehensive and reliable information on diabetes incidence, mortality rates and lack of longitudinal studies in India. In light of the scarcity of evidence in India, the objective of this study was to estimate the incidence of diabetes and its lifetime risk in an urban community of Mumbai.

**Methods:**

The research study utilized data which is extracted from the electronic medical records of beneficiaries covered under the Contributory Health Service Scheme in Mumbai. The dataset included information on 1652 beneficiaries aged 40 years and above who were non-diabetic in 2011–2012, capturing their visit dates to medical center and corresponding laboratory test results over a span ten years from January, 2012- December, 2021. Survival analysis techniques are applied to estimate the incidence of diabetes. Subsequently, the remaining life years from the life table were utilized to estimate the lifetime risk of diabetes for each gender, stratified by age group.

**Results:**

A total of 546 beneficiaries developed diabetes in ten years, yielding an unadjusted incidence rate of 5.3 cases per 1000 person-years (95% CI: 4.9- 5.8 cases/ 1000 person years). The age-adjusted lifetime risk of developing type II diabetes in this urban community is estimated to be 40.3%. Notably, males aged 40 years and above had 41.5% chances of developing diabetes in their lifetime as compared to females with a risk of 39.4%. Moreover, the remaining lifetime risk of diabetes decreased with advancing age, ranging from 26.4% among 40–44 years old to 4.2% among those age 70 years and above.

**Conclusion:**

The findings stress the significance of recognizing age specific lifetime risk and implementing early interventions to prevent or delay diabetes onset and to focus on diabetes management programs in India.

## Background

Type 2 diabetes mellitus (T2DM) accounts for 90–95% of all diagnosed diabetic cases worldwide [1]. There has been many fold increase in diabetes prevalence over the past few decades and as per the International Diabetes Federation's (IDF) latest report in 2019, around 9.3% of the world population was diabetic [2, 3]. Diabetes is the ninth leading cause of death in 2019 with an estimated 1.5 million deaths directly caused by it and is highly associated with increased morbidity, mortality, and high health care costs [4–7].

Literature has found that Asian Indians have a higher risk of developing diabetes compared to other ethnic groups [8–10]. It has been established that with an upsurge in number of early-onset diabetes cases and the increase in the life expectancy of diabetic individuals has majorly led to increase in the number of years spent with diabetes and related sufferings [11, 12]. India is regarded as the diabetes capital of world and its increasing prevalence is a cause of concern as how much this disease might impact the country in the coming years. There are a very few studies that have estimated the incidence and the lifetime risk of diabetes in India based on continuous and a longer follow-up. The incidence and prevalence do not completely capture an individual’s risk of developing diabetes during a defined period, whereas the lifetime risk, is an estimate of the cumulative risk of developing a disease during an individual’s lifespan, which is relevant for patients, clinicians, and health-care policy makers. The lifetime risk is more intuitive, easily understandable measure that estimates the length and quality of life and its effect on individuals’ life [4, 13].

The lifetime risk has been estimated for various non communicable diseases [14] like cardiovascular diseases [15–17], coronary heart disease [18, 19], cancers [20] etc., whereas a very few studies have estimated the lifetime risk for diabetes [4, 13, 21–26]. There is limited evidence of similar kind in India and potential reasons are the dearth of comprehensive and reliable data on incidence and mortality, underdiagnosis and undertreatment of diabetes, high costs associated and time-consuming longitudinal studies, and limited funding for research [27–29]. A very few of all these researches have particularly focused on lifetime risk of diabetes in India [6, 26, 30]. Previous studies have used markov simulation models, data inputs have been borrowed from other data sources, and have lacked continuous follow-up data on incident diabetes based on cohort. In particular, the study by [26] estimated the lifetime risk of diabetes using data inputs from Centre for Cardiometabolic Risk Reduction in South Asia study (CARRS) and Indian Council for Medical Research's INDIAB study to estimate the lifetime risk in a few metropolitan cities of India. Also, while studies have shown that men have a higher risk of developing type 2 diabetes compared to women, the nature of differences in the lifetime risk of diabetes between these groups is yet to be quantified. The limited statistics in Indian context on lifetime risk hinders planning of targeted interventions to reduce the burden of diabetes in India. Given the lack of evidence in terms of lifetime risk of diabetes in India, this study endeavors to quantify the lifetime risk to diabetes within an urban community in Mumbai, with a secondary goal that involves generating age and sex-specific estimates of the risk.

## Methods

### Study design and study participants

The is an observational ten-year retrospective cohort study using data extracted from the Electronic Medical Records of a Contributory Health Service Scheme (CHSS) beneficiaries located in an urban community of Mumbai. A total of 1652 beneficiaries aged 40 years and above who were non diabetic in two consecutive years 2011–2012 were followed up for a decade i.e., from January 2012 to December 2021. This study captured lifetime risk using the data on the onset of type II diabetes among these cohorts in this 10-year observation period. A detailed protocol followed for setting the CHSS linked cohort known as CHIPS cohorts, including sampling design and the selection criteria of these beneficiaries for the study including their characteristics is published elsewhere [31].

### Data

Study extracted the data on the date of visits to the hospital for various medical facilities and the results of the three of the laboratory tests related to diabetes from a hospital’s and its sixteen peripheral clinics’ Health Management Information System (HMIS). The study used the definition given by the American Diabetes Association (ADA), 2021 [32], where an individual is considered to be Diabetic if the Fasting Plasma Glucose (FPG) ≥ 126 mg/dl (7.0 mmol/l), or Postprandial Glucose (PPG) ≥ 200 mg/dl (11.1 mmol/l), or HbA1c ≥ 6.5% or that individual is on anti-diabetes medication.

### Statistical analysis and software

The study estimated the lifetime risk of diabetes, which is a crucial measure of the overall burden of a disease. It is defined as the probability of developing the disease during the remainder of an individual's lifespan. Calculation of the lifetime risk of diabetes involves the utilization of two essential variables: the age-specific incidence rate of diabetes, which denotes the new cases of diabetes per year in a particular age group, and the remaining life expectancy for that age, which defines the age range for which an individual is further expected to be at risk. The survival analysis techniques are used to estimate the age and sex specific incidence rate of diabetes. The incidence rate is calculated by dividing the number of new cases of diabetes in the span of ten-year divided by the person years (observation time) contributed by these beneficiaries starting from the date of birth till the last date of follow-up or till the date of diagnosis of diabetes, whichever occurred earlier. The incidence rate is further weighted and adjusted for the age and sex structure of the whole beneficiary’s population as a correction factor for sampling. The second component i.e., the remaining life years for each age and gender is derived from the abridged life tables from the Sample Registration System (SRS) 2012–2016 report [33].

The lifetime risk of diabetes is then calculated as:

$$\mathbf{R}\mathbf{e}\mathbf{m}\mathbf{a}\mathbf{i}\mathbf{n}\mathbf{i}\mathbf{n}\mathbf{g}\;\mathbf{L}\mathbf{R}=1-\boldsymbol{ }{\left[1-\mathbf{I}\mathbf{R}\mathbf{x}\right]}^{{\varvec{L}}{\varvec{E}}{\varvec{x}}}$$

where,

LR: Lifetime Risk.

IR_x_: Incidence Rate for the age group x to x + 5.

LE_x_: Life expectancy for the age group x to x + 5.

Similar formula has been used by the Sample Registration System for estimating the lifetime risk of maternal mortality [34]. All the analysis of this study is done in statistical software STATA version 16.0 and the Microsoft excel.

## Results

### Age sex distribution of the sample

Table 1 presents the age distribution of the total beneficiary population, categorized by sex. The data indicates that in the baseline year, approximately 56 percent of beneficiaries were 60 years old or older, of which around 28 percent of beneficiaries being 70 years old or older. Among the beneficiaries, males had a higher mean age of 59 years, whereas females had a mean age of 53 years.

**Table 1:** Age-sex distribution of cohort of non-diabetic CHSS beneficiaries followed up for 10 years (*N* = 1652), 2012

	**Overall**		**Males**		**Females**	
Age Group	Number	Proportion (%)	Number	Proportion (%)	Number	Proportion (%)
**40–44**	84	5.1	18	2.4	66	7.3
**45–49**	162	9.8	49	6.6	113	12.4
**50–54**	229	13.9	73	9.8	156	17.2
**55–59**	253	15.3	102	13.7	151	16.6
**60–64**	251	15.2	125	16.8	126	13.9
**65–69**	203	12.3	109	14.7	94	10.3
**70 + **	470	28.5	267	35.9	203	22.3
**Total**	1652	100.0	743	100.0	909	100.0
**Mean age ± SD (in years)**	55.6 (± 0.3)		58.70 (± 0.4)		53.1 (± 0.4)	

### Transition over the years

Among the 1652 individuals aged 40 years and older who were a part of the non-diabetic cohort of beneficiaries in 2012, a sum of 546 beneficiaries experienced the onset of diabetes, or developed diabetes. Within a brief period of ten years, 318 beneficiaries were no longer engaged in the study and were lost to follow up before getting diagnosed with diabetes. The remaining 788 beneficiaries continued to be tracked until the conclusion of the study's follow-up in December 2021, during which time they were consistently identified as non-diabetic.

### Incidence of diabetes and the lifetime risk

The 546 beneficiaries who developed diabetes over the period contributed to a total of 109,748 person years of observation starting from the date of birth of each beneficiary till the end of follow-up, lost to follow-up or the date of diagnosis of diabetes, whichever occurred earlier. The Table 2 shows the number of newly diagnosed cases, person years contributed by all beneficiaries including males and females over the follow-up period. Overall, the incidence rate of diabetes is found to be 5.3 cases per 1000 person years (PYs), with a median follow-up time of the 8.3 years. The results show that females contributed more person years over the period with an incidence rate of 5.6 cases per 1000PYs (Table 2).

**Table 2:** The overall lifetime risk of type II diabetes among males and females aged 40 years and above

	**Overall**	**Male**	**Female**
**Total Diabetic diagnosis (unweighted)**	546	250	296
**Observation time (years)**	109,748	49,180.2	60,567.4
**Unadjusted**			
Incidence rate (per 1000PYs)	5.56	5.44	5.64
The life expectancy at birth 2012–2016	74.2	72.6	76
Lifetime risk of Diabetes (%)	**33.9**	**32.9**	**34.9**
Lower Limit 95% CI (%)	31.7	29.5	31.9
Upper Limit 95% CI (%)	36.2	36.3	38
**Age Adjusted (for Beneficiaries Population)**			
Incidence rate	6.9	7.4	6.6
Lifetime risk (%)	**40.3**	**41.5**	**39.4**
Lower Limit 95% CI (%)	37.9	38.0	36.3
Upper Limit 95% CI (%)	42.6	45.1	42.6
**Age Adjusted (for Mumbai’s 2011 census Population)**			
Incidence rate	8.1	8.8	7.5
Lifetime risk (%)	**45.1**	**47.4**	**43.3**
Lower Limit 95% CI (%)	42.7	43.8	40.2
Upper Limit 95% CI (%)	47.5	51.0	46.6

The age adjusted lifetime risk of T2DM among the beneficiaries aged 40 years and above in this urban population is found to be 40.3%. Further, the age standardized lifetime risk of diabetes is found slightly higher among males, i.e., 41.5% as compared to 39.4% risk of developing diabetes among females. Also, adjusting for Mumbai’s 40 + population, the lifetime risk of diabetes further increased to 45.1% in the study population (Table 2).

### Age- sex differentials in the lifetime risk of diabetes

The study also estimated the remaining lifetime risk of diabetes among the beneficiaries belonging to various age groups. The results show that the lifetime risk of a beneficiary aged 40–44 years is 38.5% and with increase in age, the remaining lifetime risk of diabetes decreases to just 4% among beneficiaries aged 70 years and above. The Table 3 below shows that the remaining lifetime risk of diabetes among the beneficiaries in the urban community.

**Table 3:** The lifetime risk of diabetes in for individuals belonging to each age group in the study population

Age group	Life expectancy as per SRS 2012	Incidence rates per 1000 person years	The remaining lifetime risk of diabetes (95% CI)
**40–44**	37.1	13.01	38.5 (28.1, 48.9)
**45–49**	32.5	9.02	25.5 (18.8, 32.2)
**50–54**	28.1	7.39	18.8 (13.7, 23.8)
**55–59**	23.8	7.47	16.3 (11.8, 20.9)
**60–64**	19.7	5.35	10.0 (6.3, 13.7)
**65–69**	15.9	5.19	7.9 (4.2, 11.7)
**70 + **	12.5	3.24	4.0 (2.2, 5.7)

Similar exercise was carried out among males and females separately belonging to each age group. The Table 4 provides information about the incidence rate of diabetes and the remaining lifetime risk of diabetes among males and females aged 40 years and above. The remaining lifetime risk of diabetes is found higher among males as compared to females and the age pattern shows that after the age of 60 years, females have a higher lifetime risk of diabetes in comparison to males, though the differences are not found statistically significant. The lifetime risk of diabetes in males aged 40–44 years is 44.2% and risk among the females of the same age is 33.5%. The lifetime risk of a female aged between 60–64 years 12%, whereas, males aged 60–64 years have a 7.8% risk of developing diabetes in their remaining lifetime. Among the beneficiaries aged 70 years and above, the remaining lifetime risk of developing diabetes is around 4% among males as well as females. The table shows that the incidence rate of diabetes is higher for males than for females at all ages. The table also revealed that the remaining lifetime risk of diabetes increases with age for both males and females, an interestingly the gap between the remaining lifetime risk of diabetes among males and females narrowed with increase in age. This is because a major share of the older beneficiaries become diabetic in the mid of the study period and hence the lifetime risk decreases (Table 4). The last column of the table shows the incidence rate ratios among males and females, and reveals that the ratios are not statistically significant over the years of follow-up, with an overall chi^2^ value of 5.2 and *p* value of 0.51.

**Table 4:** The remaining lifetime risk of developing type II diabetes among males and females across age groups of the beneficiary population

	**Males**			**Females**			**Incidence rate ratios**
Age group	**Incidence rates (per 1000 person years)**	**Life expectancy as per SRS **	**The remaining lifetime risk of diabetes (95% CI)**	**Incidence rates (per 1000 person years)**	**Life expectancy as per SRS **	**The remaining lifetime risk of diabetes (95% CI)**	
**40–44**	16.1	35.9	44.2 (21.3, 67.2)	10.6	38.4	33.5 (22.1, 44.9)	0.52
**45–49**	9.4	31.4	25.7 (13.5, 37.9)	8.7	33.7	25.6 (17.5, 33.6)	0.91
**50–54**	7.5	27.2	18.5 (9.6, 27.4)	7.3	29	19.2 (13, 25.3)	0.81
**55–59**	8.2	23.1	17.2 (9.9, 24.6)	6.9	24.5	15.8 (10, 21.6)	0.81
**60–64**	4.2	19.1	7.8 (3.1, 12.5)	6.3	20.3	12 (6.3, 17.6)	1.25
**65–69**	5.1	15.5	7.7 (2.7, 12.7)	5.2	16.2	8.1 (2.6, 13.7)	1.01
**70 + **	3.4	12.2	4.1 (1.7, 6.4)	3.1	12.8	3.9 (1.2, 6.5)	1.00

### Lifetime risk of diabetes at selected terminal ages

Given the study population comprising of varying age a similar exercise carried out for beneficiaries at specific terminal ages. The Table 5 shows the lifetime risk of diabetes at a few selected terminal ages among the beneficiaries. The lifetime risk for a beneficiary aged 40 years is 21% which decreases to merely 6% among those aged 70 years. The lifetime risk is found higher among males throughout as compared to females of same age. For instance, the remaining lifetime risk of diabetes among males aged 40 years is 29.5% and among females of the same age is found to be 17% (Table 5).

**Table 5:** The Remaining Lifetime Risk of Diabetes (%) in the overall study population at terminal ages

**Overall**			
**Age**	Incidence rates per 1000 persson years	Remaining Life years	Lifetime risk (%)
**40**	6.7	35.9	21.4 (0.7, 42.2)
**50**	7.0	27.2	17.4 (6.1, 28.7)
**60**	4.6	19.2	8.5 (1.5, 15.4)
**70**	5.0	12.3	6.0 (-0.9, 12.8)
**Males**			
**40**	10.0	34.8	29.5 (-10.5, 69.5)
**50**	13.3	26.3	29.7 (3.8, 55.5)
**60**	1.9	18.7	3.5 (-2.6, 9.6)
**70**	5.0	12.0	5.8 (-3.7, 15.4)
**Females**			
**40**	5.0	37.1	17 (-6.3, 40.2)
**50**	4.5	28.0	11.9 (0.5, 23.2)
**60**	8.0	19.6	14.6 (1.3, 27.9)
**70**	5.0	12.5	6.1 (-3.7, 15.8)

## Discussion

Diabetes is a chronic condition that increases the risk of other major life-threatening diseases and with changing patterns in the urbanization and lifestyle, the prevalence of diabetes has increased in the recent few decades. There is ample of literature on the self-reported diabetes prevalence but, the incidence and prevalence do not capture an overall individual’s risk of developing diabetes. The “lifetime risk”, is an estimate of the cumulative risk of developing a disease during an individual’s lifespan [26, 35], on which the evidence is limited due to lack of reliable data on incidence and mortality.

Given dearth of evidence on lifetime risk of diabetes in India, the present study estimated the age and gender specific lifetime risk in an urban community of Mumbai with unique characteristics. The population under consideration unlike the broader population of the country, here represents a group of beneficiaries registered under the Contributory Health Service Scheme, that have uniform and universal access to healthcare services, have rigorous clinical screening of diabetes, are economically stable and have moderate to high educational qualification.

After adjusting for age, the lifetime risk of diabetes within this beneficiary population was estimated to be 40 percent. This calculation was accomplished by applying appropriate weighting and adjustments to the beneficiary population aged 40 years and older. In practical terms, this means that for every five individuals aged 40 years within the study population, an estimated two of them are projected to develop diabetes over the course of their remaining lifespan. While studies have shown that males have a higher risk of developing type 2 diabetes compared to females, the nature of differences in the lifetime risk of diabetes between these groups is yet to be quantified. The present study found that males had a higher remaining lifetime risk of diabetes as compared to females, which is in contrast with most of the past studies. Previous studies [13, 21, 26, 36, 37] have reported that females have higher lifetime risk of diabetes, which most likely reflects the longer life expectancy among females as compared to males. Although in line with the present study’s results, Turin in 2016, also found a higher lifetime risk of diabetes among males with the observation that younger males had a higher lifetime risk of diabetes than their older counterparts, which indicates the importance of early mobilization of preventive measures against the development of diabetes among males [4].

Across age groups, the remaining lifetime risk of diabetes decreased with increase in age. A beneficiary aged 40–44 years had an overall estimated risk of 38.5% of developing diabetes in his/her remaining lifetime. It was found that males aged 40–44 years had an overall remaining lifetime risk of 44.2% whereas a female of the same age had a lifetime risk of 33.5% for getting diagnosed with diabetes. A previous study in India reported the lifetime risk of diabetes among males and females at age 40 years to be 47.3% and 59.2%, respectively, and for those aged 60 years, the lifetime risk was estimated to be 37.7% for females and 27.5% for males [6, 26].

There were a few countable studies that were conducted in India on lifetime risk with which the present study’s estimates could be compared. The estimates of the present study were found to be comparatively lower in comparison to these previous studies and these variations are attributed to the differences in the age for which estimates are presented and methodologies used [6, 13, 26], or differences in how diabetes is defined and evaluated. In particular, a recently published study by Luhar and his colleagues [26] used data inputs from Centre for Cardiometabolic Risk Reduction in South Asia study (CARRS) and Indian Council for Medical Research's INDIAB study to estimate the lifetime risk of diabetes in a few metropolitan cities of India among individuals aged 20 years and above. Also, majority of these previous studies have used markov simulation models and decision trees or have borrowed data from other data sources [26, 30]. Also, according to the American Diabetes Association’s definition of diabetes, it is reported that 70 percent of individuals aged 45 years are expected to develop diabetes during their remaining (Tabák et al., 2012). Unlike these previous studies, the present study used clinical data from hospital's HMIS with continuous and a longer follow-up, hence the study adds more reliable evidence on a crucial measure to the existing literature.

Although similar cohort study outside India reported that irrespective of the age, the remaining lifetime risks did not differ by sex [38]. The age pattern in the remaining lifetime risk of developing diabetes has been observed to decrease with increase in age, and these results have been consistent with reports of lifetime risk estimation for other diseases as well [4, 13, 26, 37]. This decrease reflects the shorter remaining life expectancy and period at risk among older participants [4]. It has been pointed out that at older ages the risk of other causes of death increases and older people may not live long enough to develop diabetes. Also, people susceptible to diabetes might have developed the disease at an earlier age and went undiagnosed. On the other hand, in populations with low mortality rates, there is a tendency for the projected lifetime risk of diabetes to increase due to individuals surviving to older ages, thereby providing more time for the disease to develop and progress [21]. Previous studies have explained that because men develop diabetes at younger ages as compared to women, hence, more men than women could already have had diabetes before the start of follow-up, making women at risk of developing diabetes during the course of follow-up [36]. On the other hand, hormonal differences, lifestyle choices including, and genetic predisposition could be the factors for men to have higher lifetime risk of diabetes in this urban community.

Many previous studies have been conducted outside India on lifetime risk of Diabetes among Indian residing abroad, Asians and individuals from other nationalities, which yielded comparable results with the present study. For instance, a study from United States (US) [13] revealed that the estimated lifetime risk of diabetes for males and females born in the US in the year 2000 was 32.8% and 38.5%, respectively. The same study projected that about 4% of males and 7% of females from this birth cohort would develop diabetes by the time they reached 40 years of age [13]. Similarly, another study in US by Gregg (2014), based on data from 2000–2011, reported that the lifetime risk of diagnosed diabetes starting at age 20 was 40.2% for men and 39.6% for women [39]. This study also found a larger increase in lifetime risk of 20 percentage points in men compared to 13 percentage points in women since 1985–89 and highlighted the significant decline in mortality rates and the concomitant increase in diabetes incidence [39]. A study conducted in Canada comparing the lifetime risk of diabetes among non-First Nations and First Nations adults found that men had a higher lifetime risk of diabetes than women in the non-First Nations group, whereas women had a higher lifetime risk than men in the First Nations group, with similar age strata [4]. Another literature based on the Rotterdam Study estimated the lifetime risk of Diabetes for individuals aged 45 years to be 31.3% [38]. Another recent cohort study among Chinese people reported the lifetime risk of Diabetes to be as high as 65.9% for people with normoglycemia at age 20 years using Markov Chain Model [21]. An investigation by [24] in Brazil found that the lifetime risk of developing diabetes by the age of 80 years was 23.4% for white adults aged 35 years and 30.8% for black/brown adults of the same age. Furthermore, a study from Denmark published in 2016 by [23] found that the lifetime risk of diabetes was 24% in the population under consideration. Another research on Australian population reported the lifetime risk of developing Diabetes among 25-year-old individuals to be 38% [25]. Such a corroboration is expected as these countries mentioned above are relatively better in terms of social and economic status with better access to health care, while their counterparts studied here too has universal access to health care and a living standard which is very much above the average India situation.

As mentioned, the increased risk of diabetes has also been attributed to a combination of genetic predisposition, lifestyle factors which includes diet which is high in carbohydrates and unhealthy fats, obesity, lack of physical activity, disparities in the exposure to environmental factors and various other socio-cultural-economic factors [4, 13, 21, 40, 41]. In particular, type II diabetes in Chinese and Indian populations is characterized by earlier onset and lower adiposity compared to western populations and this trend is exacerbated by rapid urbanization and the adoption of unhealthy lifestyles [37]. As a result, it is crucial to acknowledge that these differences will inevitably influence the projected lifetime risks of developing diabetes. Studies have also shown that Asian Indians tend to develop diabetes at a younger age and at a lower body mass index (BMI) compared to other populations (41). Although, these factors were not considered in the study but are significantly related to diabetes, its incidence and lifetime risk.

The study presents both strengths and limitations. Notably, its key strength is in its extensive dataset, achieved through meticulous clinical assessments via three diagnostic methods for diabetes identification. This surpasses prior studies limited by time and cost in India. The study's strength also lies in continuous follow-up, enabling accurate person-year calculations, vital for incidence rates determination.

However, the study does have recognized limitations. The population's uniform healthcare access and comparisons with previous studies need careful consideration due to age, sex, and definition differences. Generalizing findings should be cautious given distinct diabetes trends. Additionally, unexplored factors like obesity and comorbidities could affect diabetes, but were not causally examined in this study. Acknowledging these boundaries aids in interpreting the findings accurately.

## Conclusion

In conclusion, the lifetime risk of developing diabetes in India is high. In particular, two in five beneficiaries from this urban community are expected to develop diabetes in their lifetime. The findings from the study hold important message that understanding the lifetime risk and implementing early interventions, can help in preventing or delay the onset of the diabetes in the population. Targeted public health interventions are needed to address the rising burden of diabetes in India. These interventions should focus on improving access to diabetes screening and strengthening preventive measures, raising awareness about the importance of early diagnosis and treatment and promoting healthy lifestyles.

## Data Availability

The datasets generated and/or analysed during the current study are not publicly available due to data privacy norms of the hospital under consideration, but are available from the corresponding author on reasonable request at pal3193@gmail.com, after due approvals.

## List of Abbreviations

ADA: American Diabetes Association
CARRS: Centre for Cardiometabolic Risk Reduction in South Asia study
CHIPS cohort: CHSS-IIPS cohort
CHSS: Contributory Health Service Scheme
FPG: Fasting Plasma Glucose
HbA1c: Glycated Hemoglobin
HMIS: Health Management Information System
IDF: International Diabetes Federation
INDIAB: INdia DIABetes
IR: Incidence Rate
LE: Life expectancy at age x
LR: Lifetime Risk
PPG: Postprandial Glucose
PYs: Person years
SRS: Sample Registration System
T2DM: Type 2 diabetes mellitus
